# Hospital and Institutional News

**Published:** 1919-10-04

**Authors:** 


### HOSPITAL AND INSTITUTIONAL NEWS.

### THE LATE SIR JOHN FURLEY.

Sir John Furley, C.H., C.B., whose fame was world-wide as one of the original organisers of the St. John Ambulance Association, and one of the founders of the British Red Cross Society, died on Saturday, September 27, at his residence at Oxford in his 84th year. In the year 1868 Mr. Furley became associated in a provisional committee for the establishment of a National Red Cross Society for the relief of the sick and wounded in war, similar to the societies which had already been formed in other countries. As a representative of this Committee he attended the first international conference at Berlin. Two years later, 1870, occurred the war between France and Germany and Mr. Furley went to Geneva to intimate the formation of the English branch to the International Committee of the Red Cross. On the outbreak of the South African War Sir John Furley was requested not only to design, but also to take command of the hospital train which was sent out at the suggestion of Princess Christian. Lady Furley, who was her husband's untiring and devoted helper, accompanied him to South Africa, and the first journey of the train was to Ladysmith, whence it returned to Durban with officers and men who were safely placed on board a hospital ship. During the eighteen months of Sir John's control, this train carried as patients 321 officers, 19 nursing sisters, 7,208 men, with only six deaths. When the late war broke out Sir John Furley in spite of his advanced age rendered valuable service to the Red Cross, and under his personal supervision were designed the original 25 huts, the nucleus of the great base hospital at Netley, which afterward served as models for later additions. Sir John Furley received many marks of public recognition, in this and other countries.

### RESTRICTING THE POPULATION.

Dr. Pilkington, the medical officer of health for Preston, in his annual report, refers to many of the infantile deaths as having been due to premature birth or to a condition of atrophy or debility at the time of birth, declaring that there can be little doubt but that many deaths were the result of those means employed for the prevention or limitation of families which have had such a disastrous effect upon the birth-rate and have so greatly reduced the national increase of the population. He proceeds to say that the illegal methods employed to prevent or limit the responsibilities connected with a family of children too often act in the desired direction, but not infrequently if even the main object is not attained they exercise a disastrous effect upon the health of the mother and child exposed to their influence. The honour and nobility connected with motherhood appear to be no longer required. Home life has lost its charm, and pride in household management is swamped by a feverish desire for liberty, excitement, and an unrestricted round of pleasure. Nor is this condition confined to one class of society, and the luxury formerly supposed to be confined to the wealthy is, under the stimulus of shorter hours of labour and vastly increased earnings, gradually invading the homes of the working classes. Possibly, adds Dr. Pilkington, this may to some extent be due to the large amount of responsibility as regards the upbringing of children which is now taken from the shoulders of parents, leading them to take less interest in matters which are to a large extent done for them by the State.

### DECRYING SANATORIUM TREATMENT.

Reporting to the Preston Corporation Dr. Mary Lowry, acting tuberculosis officer and assistant medical officer of health, says it has been the fashion during the last year or two to decry the usefulness of sanatorium treatment, and those who have been foremost in this criticism were no doubt those who at one time hailed sanatoria as the remedy for all cases of tuberculosis. Given early cases, a suitable sanatorium, and a sensible patient, sanatorium treatment has no doubt had uniformly good results. It cannot, however, be expected that a patient accustomed to the comparative mildness of the Preston climate can thrive and flourish in the intense cold of the north-east coast of Scotland. It must be remembered also that during the war all sanatoria have been badly handicapped by difficulty in getting food supplies, shortness of staff, and the utter impossibility of getting any improvements in the buildings carried out. As far as Preston's experience goes, sanatorium treatment has been, in the large majority of cases, very satisfactory. The disappointments have been cases which have had to return to unsatisfactory housing conditions and unsatisfactory financial conditions. The chief criticism of sanatorium treatment, in her mind, is that, carried out as it is at present, the time allowed is far too short for many cases.

### THE LAST WAR YEAR OF THE M.A.B.

Reading between the lines one would judge that the administration of the great organisation known as the Metropolitan Asylums Board was a work of considerable difficulty during the war. The M.A.B. embraces, amongst many other features, fourteen large hospitals for infectious diseases, three consumption sanatoria and sundry colonies for epileptics, training-ships, and casual wards. The admissions last year to the hospitals alone were over 18,000. The ambulance service dealt with 31,000 removals of infectious patients, and the mileage run by the vehicles was 472,189. To deal with all this service for the public without the simplifying element of contracts for food and materials must have almost doubled the administrative work and responsibility. It was not merely a cost of buying food, for the works carried out under the direction of the engineer-in-chief represented a cost on building and engineering work and repairs of £13,500. As to general supplies, these were the concern of a special committee, who had frequently to make very large purchases of goods in bulk, available on the spot and only open for sale subject to immediate acceptance, being sales practically to the first comer. Periodical contracts became a rare exception and more in the nature of securing sources of supply, the terms and conditions of which were subject to mutual agreement from time to time.

### THE CASE AGAINST MR. R. V BANKES.

There has been a growing tendency of late for public authorities to condemn the magistrates for what they consider the inadequate fines imposed on vendors of adulterated articles. For instance, out of 118 samples of milk analysed in two months in Lambeth, 19 were found to be adulterated. In two cases the enormous amount of over 20 per cent, of added water was found. When the magistrate inflicts only a pound fine, no wonder there are complaints, for the vendor can promptly recuperate himself by the sale of greatly adulterated milk. Mr. Ralph V. Bankes, the magistrate at the South-Western Police Court, who has been attacked by the Local Food Control Committee, points out that the louder the clamour is the more important it is that the magistrate shall be impartial. Justice is never worse administered than under public pressure. Undue punishment only occasions resentment. Some of the defendants were poor people, and to treat all cases alike appears to him to be a denial of justice. Unfortunately, there is an enormous amount of adulteration going on, and in many cases it is difficult to obtain evidence. That is why local authorities feel aggrieved when what they consider inadequate fines are inflicted. In the cases of milk adulteration warranties are produced, which protect the vendor, and until this vicious system, alike condemned by the magistrates and local authorities, is abolished, there is slight chance of getting a purer milk supply. So the case against Mr. Bankes' argument is considerable.

### THE TEST OF THE PATIENT.

A dreary correspondence has been proceeding in the *Portsmouth Evening News* on the rival merits of the voluntary and the municipal hospital. The trouble is not that the former lacks overwhelming arguments, but that the men who represent them and know the facts of both systems (the municipalisers and crude socialists as a rule who have not passed beyond the doctrinaire standard of the debating society) have been infected by the bureaucratic wave of opinion which always follows in the wake of war, and consequently do not speak with conviction. We therefore read without comment a controversy as stale as it is fashionable until we came across the one letter, signed by C. A. Scott Ridout, which attempts to take an impartial view of both sides. The writer, after alluding to the stock arguments of either side, sums up by stating that the issue depends on "how the institution will be run." This is a welcome return to common-sense because it implies, though it should emphasise the fact, that the test of any hospital is neither its freedom from financial anxiety, nor even the number of its beds, but, once and for all, the welfare of the patient. We need not repeat the explanation of this test, for an old test though it is to us, it was the subject of an article in The Hospital of September 27 entitled, "The Voluntary Hospital Essential to Public Safety." In response to a request from an Irish correspondent we lately published an article on "The Meaning of Efficiency." If the readers of the *Portsmouth Evening News* would study both articles we believe that all who are capable of reasoning will see clearly why the voluntary system must be maintained.

### FINANCIAL AID FOR CONSUMPTIVES.

The Camberwell Interim Tuberculosis Care Committee has been in communication with the Ministry of Health relative to the provision of financial assistance to persons suffering from tuberculosis. The Committee is anxious to raise a fund so that it may be in a position to give direct financial help to patients recommended to it by the local dispensary, local doctors, and certain hospitals, instead of being obliged, as at present, to refer these patients to such societies as the Charity Organisation Society or the Parochial Relief Fund. The Committee urged that in many cases financial help would be invaluable, and that, for instance, the provision of water beds and bedside appliances would make all the difference to the comfort of a patient who was an advanced case. The Ministry of Health informs the Committee that it is not at present empowered to make grants in aid of the general expenditure of a Tuberculosis Care Committee, but as regards the provision of water beds, etc., it is competent for a borough council to incur reasonable expenses in the provision of such appliances for use in special cases. If the Care Committee were to purchase such appliances it might, in the opinion of the Ministry, approach the borough council for a contribution to such expenditure. The Ministry has also informed the Camberwell Borough Council that any contribution so made can be included in the Council's tuberculosis maintenance account, with a view to one-half of the expenditure being repaid by the Ministry.

### THE FINANCIAL PROBLEM IN A NUTSHELL.

The Coventry and Warwickshire Hospital is badly in need of £12,000 to meet its debts, and for a sum to replace the War Office grant which will not be received next year. In reviewing the chances of obtaining these sums, the local papers devote themselves rather to enlarging on the difficulties than to explaining and remedying them. The chief difficulty alleged is that "there is a narrower margin" from which to draw "charitable contributions"; and in support of this the *Coventry Herald* contends that "the incomes of tradesmen and middle-class people generally have felt and do feel the strain," and so forth. Frankly, we can accept neither excuse. The margin is not narrower; but the people who formed the margin from which hospitals obtained their supporters before the war are different. In this connection take the second point, that "tradesmen and middleclass people generally" have been pinched too much to give to charities. The truth is that established tradesmen have grown richer, that new tradesmen have become suddenly prosperous. It is the middle-class which had a little property and neither the opportunity nor the appetite for profiteering which has suffered. It is these innocents who formed a body of hospital subscribers. Wealth has changed hands. The real problem is to educate its new possessors, and to teach them that generosity to charity is the first responsibility that they must shoulder, now that the chance to make money has come to them.

### THE NEW SUSSEX HOSPITAL.

We have already predicted that in the provinces, except, perhaps, in university towns, women doctors will not unduly feel the pinch of competition. For the provinces are now beginning to establish hospitals staffed by women, such as the New Sussex Hospital for women and children at Brighton, which is on its way to working order. To further the scheme, already described in The Hospital, a meeting was held on September 17, presided over by Councillor Mrs. Chapman, and addressed by the Bishop of Lewes, whose official residence is at Hove. He reminded the audience that 60,000 had joined the V.A.D.s., and that women doctors had justified themselves during the war; though two years' work had been necessary to convince the Government of their capacity and endurance. Started in 1912 by Dr. Martindale and a staff of women, the New Sussex Hospital last year treated 242 in-patients, and 9,000 out-patients. The gift of £5,000 from the Howard Trustees encouraged the hospital's supporters to expand the institution from the three small houses in which it was formed. Windlesham House, in Norfolk Terrace, had been bought, and if £50,000 can be raised, an up-to-date hospital of 50 beds can be provided. Paying patients will be admitted, and the secretary, Miss R. E. Green is hoping for support from Worthing, which has been invited to form a support committee of its own.

### A CRUMB OF COMFORT FOR OUR CROAKERS.

Weak-minded hospital managers and secretaries who are beaten by their job, have been seriously dismayed by the withdrawal of the War Office capitation grant with the cessation of military patients. The temptation of the Government grant has been strong to these men, and a good deal of shamefaced hope is secretly entertained that Dr. Addison and the Ministry of Health may be induced, in some new form, to re-issue it. The croakers appear in the lay newspapers, and confess their own shortcomings under the plea of "hospital bankruptcy." It is the old story of incompetence clamouring in public, and seeking to hide itself in a general confession of failure. When we listen to these people, we are ashamed that the voluntary system should so much as seem to be in their hands; though we welcome their self-confession of their worthlessness. There is, however, this crumb of consolation for them. If their wish is granted, and the large measure of State aid for which they are secretly working is accorded, they will find that their services will be no longer wanted, for the first thing which the Ministry of Health, under such circumstances, will do is to clear them all out, and replace them by medical superintendents. It has been done in America, and, sooner or later, it will be done here. Dr. Addison, with his professional bias, is not likely to be recalcitrant to the strong arguments in favour of this change. Our only regret is that it should be left to the Minister of Health to do that which the hospitals ought to do for themselves—namely, free the hospitals from their incompetents.

### BRISTOL v. RED CROSS GRANTS.

We are not surprised that, according to the *Bristol Times and Mirror*, "much surprise has been expressed that, up to the present, no grant has been made to Bristol from the surplus funds which are in the hands of the Joint War Committee of the British Red Cross Committee and the Order of St. John." In going through the list, which was the subject of the first leading article in The Hospital of September 20, we noticed several remarkable omissions. King Edward VII. Hospital, Cardiff, appeared to receive no grant, nor did the Worcester Royal Infirmary, though a handsome grant was made to another institution in the same county. There were other apparent omissions, but these will serve, and Bristol seems to be the first centre to complain. We foresaw these complaints, but the explanation, as we stated at the time, is that "the county directors of six English counties, the West Riding of Yorkshire, and one Welsh county appear not to have applied in accordance with the conditions under which the grants have been made." We must assume, therefore, in the absence of evidence to the contrary, that the county of which Bristol is the industrial capital, was one of these. Who is the county director, and if he did not apply in accordance with the conditions, why not? To get an answer to these questions will be more fruitful than to indulge in complaints. We invite the *Bristol Times and Mirror* to discover and report the truth.

### "A NEW CHANNEL OF ADVERTISING."

In reply to a suggestion referred to in our Notes under this heading on September 20, a hospital secretary writes: "We have advertised in parish magazines for years," and he goes on to mention a seaside resort, a northern spa, and to say that his hospital's advertisements in London parish magazines have been confined to those in the West End, as is natural. References to prices of advertising always produce interesting correspondence. We welcome the attention being devoted by secretaries to this branch of publicity, directly by "announcements" and indirectly by contributions to the news columns of the lay press. Though these are important, the second is in principle the better, partly because all powerful influence is indirect, but mainly, as we have insisted so often, because "the only finally decisive advertisement" for a voluntary hospital is the value of its work. To be valuable it must be good and vigorous, and it is the atmosphere in which such work is done that excites interest, attracts friends, and alone ensures permanent support.

### NEW HEALTH POWERS.

Swansea Corporation Health Committee has decided to seek further health powers enabling the Corporation (1) to repair defective drains and recover the cost from the owner; (2) require buildings to be disinfected in order to prevent the spread of tuberculosis; (3) to require food-storage accommodation to be provided in existing houses where practicable; (4) to impose penalties on persons suffering from infectious disease exposing themselves in public places; (5) to close Sunday schools during prevalence of infectious disease; (6) to require persons suffering from pulmonary tuberculosis to be removed to a hospital; (7) to prevent the contamination of sausages and similar goods; and (8) to prevent rag and bone dealers from selling food at their premises or from their carts, etc. This is a formidable list, but, as the Town Clerk points out, there are precedents in local Acts for all the new powers which Swansea seeks.

### A NURSES' CHOIR.

Following an appeal made by the General Superintendent and Secretary (Mr. Lucas) of the Royal Albert Edward Infirmary, Wigan, for the endowment of beds and cots as memorials of those who fell in the war, those associated with a Red Cross organisation for the local hospitals during the war decided to hold a fête. At this some £1,030 were raised. A happy sequel was the dedication of the bed at an open-air service which was held in the beautiful grounds of the infirmary. The ceremony was performed by the Bishop of Liverpool (Dr. Chavasse), who was accompanied by representatives of the town's various denominations and members of the infirmary board of management. There was a choir of some seventy voices, including most of the members of the hospital's nursing staff, and the audience numbered nearly 1,000.

### THE COLLIERS' SPORTS.

The colliers of Wigan and district are keen sportsmen. They most enjoy and follow football, boxing, whippet-racing, pigeon-flying, and bowling. In connection with the last the superintendent, Mr. Hedley Lucas, has attracted another source of income for the under-mentioned infirmary. The response has been such that many of the bowling clubs are organising annual matches for the benefit of the Royal Albert Edward Infirmary, Wigan, and in a little over a year about £200 has resulted, and probably some £50 will be received before the close of the present season. Moreover, practical interest in the hospital's welfare has been manifested in other ways as the outcome of the foregoing.

### THE FAITHFUL FEW AND PROFITEERS.

That voluntary hospitals have been supported by enthusiasm rather than by numbers is well established. It is, indeed, a consolation to know that the class which supports them is loyal even though it might well be more numerous. Otherwise they could not have continued on a voluntary basis. But if we want to know how the generous section of the public has been affected by the war, one can learn by some remarks made by Mr. E. W. Stead, of Cumberland Infirmary. First note that the class which supports hospitals is one in that support, but in other respects is drawn from every rank in society. If their generosity alone is the common character of hospital supporters, we may well ask how generosity has been affected by the war. Here Mr. Stead's experience is interesting. He told the governors lately that Cumberland Infirmary had a "faithful body" of subscribers, who stick to their hospital from year to year, and who respond to any special effort. Well, lately Mr. Stead looked through the list of these loyal supporters, mainly local people of whom he would know. After going through their names, he could not see, he said, that "any of these subscribers had benefited by the war." What a tribute to the influence of hospitals on those who have once come in contact with them. It is those who have not come into contact with hospitals who have benefited by the war—some enormously. The problem is to interest them.

### MASSAGE ESTABLISHMENTS.

Establishments for massage or special treatment continue to be opened, and last quarter twenty-five more were registered in London by the L.C.C. The number of such establishments now registered is 1,088. As a result of experience in the administration of these places the Council has found that certain amendments of the existing law are necessary, and the Public Control Committee will bring forward some proposals when the L.C.C. resumes in October.

### THIS WEEK'S DRUG MARKET.

Business in the drug and chemical markets, like all other markets, is naturally affected by the strike, but not to anything like the extent that pessimists would have thought probable. Business men generally are keeping their heads, and are not likely to lose them, even should the strike be more prolonged than is generally anticipated. Orders are being placed much as usual with the knowledge that except the small parcels required for immediate use, there is likely to be considerable delay in transit. Prices are on the whole well maintained; any easier tendency that may be noticed in the prices of here and there a few articles is not due to the strike. It is obvious, however, that the delay in the arrival on the market of drugs of which stocks are low is likely to lead to temporarily higher prices for such drugs. There is no change in the position of quinine. The value of potassium bromide is firmly maintained. Japanese refined camphor, Japanese peppermint oil, menthol and cloves have an upward price tendency. At the recent public sale of drugs in Mincing Lane, senna leaves, myrrh and Carthagena ipecacuanha realised good prices.

